# Infected Completely Isolated Enteric Duplication Cyst Management with Percutaneous Drainage and Surgical Excision after Retreat of Infection: A Case Report

**DOI:** 10.1155/2013/108126

**Published:** 2013-02-07

**Authors:** Neofytou Kyriakos, Chysochos Andreas, Sammouti Elena, Andreou Charalampos, Georgiou Chrisanthos

**Affiliations:** ^1^Department of Surgery, Nicosia Government Hospital, Palaios Dromos Lefkosias-Lemesou No. 215, Strovolos, 2029 Nicosia, Cyprus; ^2^Department of Radiology, Nicosia Government Hospital, Palaios Dromos Lefkosias-Lemesou, No. 215, Strovolos, 2029 Nicosia, Cyprus

## Abstract

Duplication cysts (DCs) of alimentary tract are rare congenital malformations. They are firmly attached to the wall of the gastrointestinal tract and they are supplied by surrounding mesenteric blood vessels. More than 80% of cases occur before the age of two years and only a minority of cases present in adulthood. “Completely isolated duplication” of the alimentary tract is an extremely rare variety of gastrointestinal duplications. They have gastrointestinal epithelial and wall characteristics without an anatomic association with the alimentary tract. Their main characteristic is that they have their own blood supply. A 20-year-old male was admitted to our department with symptoms persisting for a period of one week prior to admission, which included abdominal pain, fever, and a palpable abdominal mass. CT revealed an unexplained intraperitoneal abscess. This case represents a rare clinical example of infected isolated duplication cyst managed with percutaneous drainage and surgical excision of the cyst 3 weeks later. To the best of our knowledge, this is the first reported case to use this approach.

## 1. Introduction

Gastrointestinal duplications (GDs) are rare congenital malformations that may vary greatly in presentation, size, location, and symptoms. They are either cystic or tubular. Cystic duplications rarely communicate with the intestinal lumen. In contrast, tubular duplications usually communicate with the intestinal lumen. In almost half of the cases, duplication cysts are associated with other malformations, mainly located in the vertebrae and genitourinary track. They share a common blood supply with the adjacent bowel wall and they tend to be located on the mesenteric aspect of the alimentary canal.

GDs originate anywhere along the alimentary tract from the tongue to the anus, with small bowel and particularly ileum being the most common location for these entities [1–3]. The colon is the least common site of enteric duplication, and only 15% of enteric duplications originate from this.

More than 80% of cases present before the age of two years [1, 2, 4]. Colonic duplications are encountered in adults in only a few cases. In general, presentation may include vague abdominal pain, distention and complications such as obstruction, bleeding, perforation, or malignancy.

Completely isolated duplication cyst is an extremely rare variety of gastrointestinal duplications and very few cases have been reported. They do not communicate with the normal bowel and their main characteristic is the fact that they have their own blood supply [5–7].

Herein we report an adult male with an infected completely isolated duplication cyst.

## 2. Case Report 

A 20-year-old male was admitted to our department with one-week history of abdominal pain and fever. The previous medical history was unremarkable. His body temperature was 38.7°C and physical examination revealed a palpable, painful mass occupying the right abdomen. Routine laboratory studies showed elevated WBC to be 16500 × 10^9^/L, C-reactive protein 40 mg/dL, whereas all other findings were normal.

**Figure 1:**
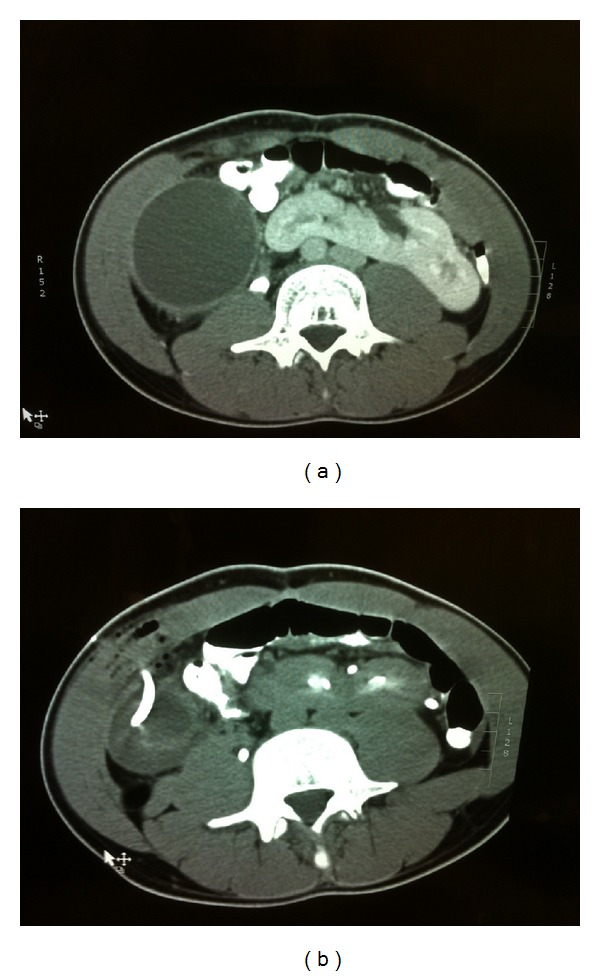
(a) Thickened wall cyst and horseshoe kidney. (b) Drainage of the cyst.

CT scan revealed a cystic mass measuring 25 × 10 × 7 cm, occupying the right abdomen, and extending from the lower surface of the right lobe of the liver to the pelvis. The cyst was laterally of ascending colon. The wall of the cyst was thickened and a small amount of free peritoneal fluid was presented. An incidental finding was the existence of horseshoe kidney (Figure 1). 

A widely spectrum antibiotic (Piperacillin/tazobactam) was administered intravenously and a CT-guided percutaneous drainage of the cyst was decided. 1200 mL pus was aspirated and two drainage tubes were left in place (Figure 1). The drainage from the tubes gradually reduced and stopped after one week. At the end of this period, the patient was asymptomatic and afebrile. WBC and CRP returned to normal values. 

The drainage tubes were removed; the patient was discharged with per os administration of Amoxicillin/clavulanic acid for one week. The patient was scheduled for exploratory laparotomy two weeks later. 

**Figure 2:**
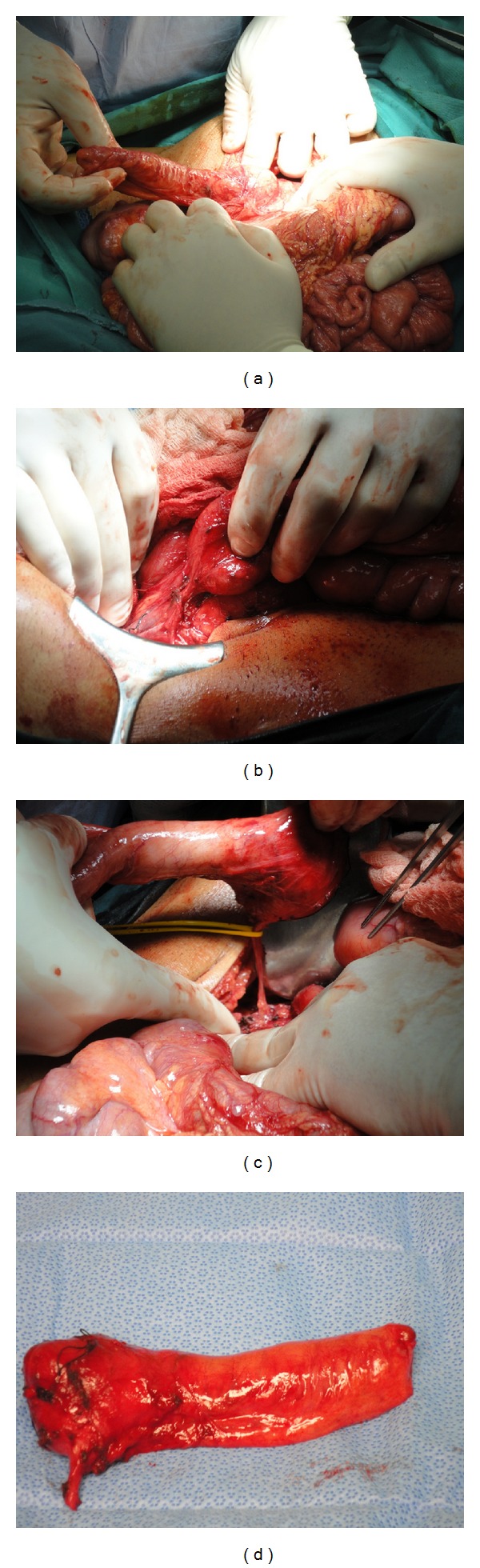
(a) Bowel-like cyst with no connection with the gastrointestinal tract. (b) Retroperitoneal attachment of the cyst. (c) Feeding vessels to the cyst. (d) The excised cyst.

At laparotomy there was a bowel-like mass, measuring 7 × 4 cm in size, that did not connect with the gastrointestinal tract and its mesentery (Figure 2). The cystic mass was free, lateral of ascending colon and only its basis was attached to posterior peritoneum. Within this retroperitoneal attachment, the feeding vessels to the cyst were dissected and the entire cyst was excised without difficulty. The final histopathological outcomes were consistent with an enteric duplication cyst with colonic mucosal lining. 

The patient was discharged from the hospital 3 days later and remained asymptomatic throughout a 1-year follow-up period.

## 3. Discussion

Alimentary duplication cysts were first described by Wendel in 1911. They are uncommon congenital lesions with a reported incidence of 1 in 4500 [2]. By definition, they are located in or adjacent to the wall of part of the gastrointestinal tract, they have smooth muscle in their walls, and they contain some type of intestinal mucosal layer within the lumen. The mucosal lining within alimentary tract duplications may display components of several different types of GI tract or respiratory tract mucosa [8].

In our case, the histopathologic examination revealed colonic mucosal lining. The cystic-like lesion's wall was similar to large bowel wall, infiltrated by chronic nonspecific inflammation. 

Enteric duplication cysts usually share a common wall with the normal intestine and have a common blood supply. “Isolated duplication” or “completely isolated duplication” of the alimentary are an extremely rare variety of gastrointestinal duplications. They are congenital cystic lesions having gastrointestinal epithelial and wall characteristics without an anatomic association with the alimentary tract [5, 9–13].

In our case, the isolated duplication cyst had no anatomic association with the alimentary tract. There was no luminal communication with the adjacent ascending colon and the cystic mass had a separate vascular pedicle which debouched from retroperitoneal space. 

 More than 80% of enteric duplications present before the age of two years, usually with intestinal obstruction or a palpable mass [2, 4]. Most of the adult intestinal duplications are asymptomatic and remain undiagnosed for years. Acute onset or chronic complaints or both are possible presentations in adults. Common findings are palpable mass and bowel obstruction [8]. Rare presentations are bleeding from ulceration within the duplication, fistula formation with nearby structures [14], and infection of the cyst. Also it has been reported that enteric duplication may be the site of adenocarcinoma [15].

In our case, the first presentation was an unexplained intraperitoneal abscess. This drove us to affront it in the same way we usually treat intra-abdominal abscesses. Infection resolved with transcutaneous drainage in combination with intravenous and later per os antibiotics. During exploratory laparotomy, there were no signs of infection and the entire cyst was excised easily. 

In almost half of the cases, duplication cysts are associated with other malformations, mainly located in the vertebrae and genitourinary track. Horseshoe kidney was present in our case. 

Imaging studies like CT, MRI, ultrasound, colonoscopy, barium meal, barium enema, and recently endoscopic ultrasound [16] have been utilized for the evaluation of enteric duplications. Cyst complications make the differential diagnosis of these lesions from mesenteric or omental cysts difficult. 

## 4. Conclusion

Infected completely isolated enteric duplication should be considered in the differential diagnosis of unexplained intraperitoneal abscess. Percutaneous drainage of these “abscesses” is safe and effective during acute phase. Excision of the cyst is much easier and safer after the retreat of infection.
